# Means to an end: teleological bias in moral reasoning

**DOI:** 10.3389/fpsyg.2025.1380048

**Published:** 2025-06-27

**Authors:** Eloise Davenport, John D. Coley

**Affiliations:** Department of Psychology, Conceptual Organization, Reasoning, and Education Laboratory (CORE Lab), Northeastern University, Boston, MA, United States

**Keywords:** promiscuous teleology, moral judgments, causal reasoning and explanation, perceived intention, moral reasoning, cognitive load, outcome bias, accidental harm

## Abstract

No model to date has integrated findings from teleological explanation with findings from moral reasoning to explore an underlying mechanism of moral cognition. We hypothesize that a preference for teleology, whereby consequences are assumed to be intentional, can explain instances where adults make judgments that seemingly neglect to account for intent. Across two studies, we investigated whether manipulating teleological reasoning influences moral judgment. 291 participants were evaluated in a 2 × 2 experimental design to assess the effects of teleology priming on adults’ endorsement of teleological misconceptions and moral judgments. Results provide some evidence that teleological reasoning influences moral judgment, but the findings are limited, context-dependent, and suggest that teleology is unlikely to be a strong influence in the explanation of outcome-based moral judgments.

## 1 Introduction

Intentions weigh heavily on the moral judgments of adults—so much so that intent to harm (*mens rea*) is a foundational tenet of American criminal law. Blackstone (1962) summarized, “An unwarrantable act without a vicious will is no crime at all. So that to constitute a crime against human laws, there must be, first, a vicious will; and, secondly, an unlawful act consequent upon such vicious will.” The law reflects a duality of consideration in which intentions are paramount, and reasoning about outcomes is separate and secondary.

A similar pattern can be observed in explanations of human artifacts and the natural world, whereby people use causal reasoning based on the assumption of a goal, purpose, or function—or teleology. The answer to why trees produce oxygen, for example, may be explained both through physical/mechanical explanations or through teleological explanations. On the one hand, trees produce oxygen as a byproduct of photosynthesis. On the other hand, it may be tempting to say trees produce oxygen so that animals can breathe. When asked this question, most people would likely offer the latter response. Even when an entity’s material cause is known, people often explain its existence with goal-based influences (Banerjee and Bloom, 2014; Kampourakis, 2020; Kelemen, 1999a,b; Kelemen and Rosset, 2009; Legare et al., 2012; Legare and Gelman, 2008; Legare and Visala, 2011; Lupfer et al., 1992; Lupfer et al., 1994; Lupfer et al., 1996; Stern et al., 2018; Weeks and Lupfer, 2000; Woolley et al., 2011).

Given this strong focus on intentionality, an important question arises: What drives people to assume intentions behind human outcomes? And why do people similarly assume intention or purpose behind an entity’s existence? An object or phenomenon is not always intentionally designed or created with purpose, just as the outcome an agent causes is not always intended. Sometimes, physical outcomes and the mechanisms by which they come about are not intuitively connected—goals and ends are not necessarily linked.

Prior research has proposed multiple explanations for the differential influence of outcomes and intentions on adults’ moral judgments and why people sometimes judge actions based on their consequences rather than the agent’s intentions. One major account is the *outcome bias*, which suggests that people judge an action as more morally wrong when it results in a bad outcome, regardless of the actor’s intent (Baron and Hershey, 1988). This bias implies that people are disproportionately influenced by the tangible consequences of an action, even when they explicitly recognize that the agent did not intend harm. Another key explanation is *negligence-based reasoning*, which posits that when a harmful outcome occurs, people often infer that the actor was careless or negligent, even if no explicit evidence of mishandling is present (Nobes and Martin, 2022; Nobes et al., 2023). Rather than assuming the actor intended harm, this perspective suggests that observers interpret negative consequences as indirect evidence of a failure to act responsibly. Others, still, generally support the negligence account but interpret consequences in terms of *hindsight bias*, which leads people to overestimate the predictability of an outcome after it has occurred, making them more likely to attribute responsibility to the actor in retrospect (Kneer and Skoczen, 2023; Margoni et al., 2019, 2023). In such cases, individuals may unconsciously assume that the actor “should have known” the outcome was likely, even if this knowledge was unavailable at the time of action. We propose that teleological reasoning could be another factor contributing to how outcomes and intentions are weighed in moral judgement.

### 1.1 Teleological bias and moral judgment

Teleological explanations constrain perceptions of why events and objects occur (see Dennett, 1978). For example, we assume that human artifacts, such as pens or coffee mugs, are intentionally designed to fulfill a purpose (Kelemen, 1999a,b). But we also frequently make misstatements like, “germs exist to cause disease.” Children are especially “promiscuous” teleologists, likely to construe evolutionary and biological phenomena in teleological terms (Kelemen, 1999a,b). But research suggests that teleological reasoning is not limited to children; adults and even experts also exhibit teleological biases, particularly under cognitive load. For example, studies have shown that when adults are under time pressure, they are more likely to revert to teleological explanations, even in domains where such explanations are inappropriate (Kelemen et al., 2013; Coley et al., 2017; Stern et al., 2018). This suggests that teleological reasoning may be a cognitive default that resurfaces when cognitive resources are constrained.

In moral reasoning, this reemergence of teleological thinking under cognitive load could explain why adults sometimes make outcome-driven moral judgments that seem to neglect intentions, especially in more complex scenarios. This pattern is particularly evident in cases of accidental harm, where outcomes occur and causation is implied, but there is no malicious intent. Some researchers argue that cognitive load engenders a pattern of moral judgment that is more focused on causation than intent, leading adults to harshly judge accidental harm-doers, similar to childlike moral judgments (Buon et al., 2013a,b). Alternatively, we propose that under cognitive load, adults, like children, necessarily tie intentions to outcome cues, unable to judge them separately. Some evidence shows that cognitive load is additionally specific to judgments of moral wrongness, but not judgments of deserved punishment (Martin et al., 2021a,b). This aligns with Cushman’s (2008) and (2013) dual-process model of moral judgment, wherein permissibility judgments rely primarily on the actor’s intent, and punishment judgments are more strongly influenced by outcomes. Given this framework, cognitive load should have the greatest influence over moral permissibility judgments in accidental harm scenarios.

While we propose that teleological reasoning may also contribute to patterns of moral judgment—by implicitly linking outcomes with assumed intentionality—we do not believe that it is an alternative explanation to those mentioned above (e.g., outcome bias and hindsight bias). Rather, we hypothesize that teleological reasoning is one contributing factor. The outcome effect in moral judgment is complex and likely arises from a combination of cognitive biases, retrospective inference errors, and assumptions about responsibility, rather than a single explanatory framework. A more comprehensive understanding of how these factors interact could help clarify the extent to which teleological reasoning contributes uniquely to moral judgments, relative to these well-documented cognitive mechanisms. Regardless, distinguishing outcomes from intentions, and determining how this distinction emerges conceptually, is paramount to better understanding the curious ways we explain the world around us.

## 2 Study 1

In Study 1, we investigated the influence of teleological priming and time pressure on moral evaluation. Specifically, we sought to prime participants to think teleologically and subsequently asked them to judge culpability in accidental or attempted harm scenarios. By leveraging scenarios in which intentions and outcomes are misaligned, we could interpret moral judgments as either intent-based or outcome-driven. In an attempted harm scenario, for instance, an actor intends to cause harm but fails to do so. Therefore, differentiating intention from outcome, as expected under normal conditions, would lead one to make an “intent-based” judgment, condemning the actor based on their malicious intent. Conversely, the same attempted harm scenario presented to someone primed to think teleologically and therefore assume intentions as inherent explanations for consequences, would lead one to make an “outcome-based” judgment— letting the actor off the hook because no harm was caused. Importantly, we do not argue that the “outcome-based” judgment outright ignores the intentions of an actor. Rather, it assumes aligned intentions behind a consequence, thus appearing to only consider the outcome. Using scenarios in which intentions and consequences are misaligned, allows us to distinguish between judgments that considered intentions and outcomes separately (“intent-based”), and those which did not (“outcome-based”).

Participants were assigned randomly to the experimental group or to the control group, which received a neutral “priming” task instead of a teleology priming task. Each group was then further randomized into speeded or delayed conditions, with participants in the speeded condition completing the moral judgment task as well as a teleology endorsement task under time pressure.

Because our argument requires an underlying understanding of intentionality at the core of moral reasoning and teleological explanation as considered here, we wanted to rule out mentalizing capacity, or the ability to think about the thoughts, goals, and intentions of others, as a sufficient mechanism to explain misattribution of intent in moral and teleological observations. If mentalizing abilities allow us to correctly infer the intentions of others, then individuals with better mentalizing abilities should be less teleological and make more intent-based moral judgments. To test this, we included a Theory of Mind task at the end of Study 1. Of particular interest is if Theory of Mind capacity relates similarly to moral judgments and teleological endorsements.

We explore the following hypotheses:

*H1*: Teleological reasoning influences adults’ moral judgments. When primed to think teleologically, adults will make more outcome-driven moral judgments, and will be more likely to assume that intentions correspond with outcomes. Conversely, reducing teleological thinking should lead to more intent-based judgments.

*H2*: Cognitive load reduces adults’ ability to reason separately about intentions and outcomes, forcing the teleological intuition that consequences necessarily imply intentions. Time pressure should therefore increase adults’ endorsement of teleological misconceptions and lead to more outcome-driven moral judgments, regardless of priming group.

### 2.1 Method

#### 2.1.1 Participants

Participants were 215 Northeastern University undergraduate psychology students (155 women, 55 men) recruited for course credit. All participants were native English speakers. 58 respondents were excluded for failing attention checks (described below) or incompletion, leaving us with a sample of 157 participants included in the analyses. Racial demographic information was provided by 100% of participants, of which 40% self-reported as white, 22% as East Asian, 16% as multiracial, 8% as South Asian, 4.5% as Hispanic, 4% as Black, 3% as Southeast Asian, and 1% as other. Less than 1% of participants identified as Middle Eastern. The demographics of this sample are not representative of the United States at large as of 2022 (U.S. Census Bureau, 2022).

#### 2.1.2 Materials and procedure

Study 1 employed a 2 (Priming: Teleology, Control) x 2 (Time Pressure: Speeded, Delayed) design. Participants completed a Qualtrics survey with four tasks in which they read excerpts, scenarios, statements, and vignettes and responded to them.

##### 2.1.2.1 Priming task

Participants in the experimental group began the survey with a teleology prime in which they read a short text defining teleology and explaining how it is used in appropriate and inappropriate contexts (see Appendix 1 for full text). In order to actively engage teleological thinking, we then asked participants to come up with explicit teleological explanations (i.e., “Why are ears of corn wrapped in husks?”; “Why do candles have wicks?”; “Why do humans make art?”). Because the purpose of this active prime was solely to induce teleological thinking, we were not concerned with the content or accuracy of responses to these prompts. Participants in the control group received a reading and comprehension questions adapted from the GRE bank that were matched for length and difficulty, but were absent of any “cause and effect” content (see Appendix 1) (Educational Testing Services, 2017).

##### 2.1.2.2 Moral judgment task

Immediately following the priming task, all participants were presented with two pairs of scenarios adapted from Young et al. (2007). One pair described a situation in which two friends touring a chemical plant have coffee together. In the “attempted harm” version of this story, one friend believes a white powder on the counter near the coffee is a toxic substance, but really, it is sugar. The actor puts the substance in her friend’s coffee intending harm, but her friend drinks the coffee and is fine. Conversely, in the “accidental harm” scenario, the actor believes the white powder on the counter is sugar, but really, it is poison. They put it in their friend’s coffee, the friend drinks it, and they die. A separate story context involved two friends kayaking in jellyfish-infested waters.

Four versions of each story context were created to account for each quadrant of the intent-outcome matrix (intentional harm, accidental harm, attempted/failed harm, benign/neutral). Each participant received two versions of each scenario, totalling four trials per participant. The versions seen per scenario differed in only intention or outcome, not both. The version pairs for each scenario were randomized and counterbalanced across participants. Altogether, this ensured that every participant saw one scenario of attempted harm and one scenario of accidental harm, which were the scenarios of interest. Each participant, therefore, had two trials in which intentions and outcomes were misaligned.

Participants responded to an attention check to ensure they understood the outcomes caused by the actor in the story (e.g., “Did Janet’s neighbor get stung by jellyfish?”), as well as an intentionality check (“Did Janet mean for her neighbor to get stung by jellyfish?”). Then, participants responded to binary punishability (“Do you think Janet should be punished? Yes/No″) and Likert-style permissibility (“Janet telling her neighbor to swim was: Forbidden/Neutral/Permissible”) measures of moral evaluation.

##### 2.1.2.3 Teleology endorsement task

After the moral judgment task, participants responded to a series of teleological misconception (test) sentences about biological and nonbiological phenomena, adapted from Kelemen et al. (2013). Nine test sentences, along with eleven true teleological and ten false teleological control sentences, were presented to participants in random order (see Appendix 2 for all statements).

##### 2.1.2.4 Time pressure

For both the Moral Judgement and Teleology Endorsement tasks, participants were randomly assigned to either a speeded or delayed condition. Those in the speeded condition read each moral scenario and answered the attention checks in the Moral Judgment task at their own pace but were required to respond within 2 s after the presentation of prompts for both tasks (i.e., punishment and permissibility questions for Moral Judgement, sentences for Teleology Endorsement). Participants in the delayed condition also read the scenarios at their own pace, but were forced to wait 10 s after the presentation of prompts for both tasks before they could respond and move to the next item.

##### 2.1.2.5 Theory of mind task

All participants responded to a series of twenty vignettes based on Dodell-Feder et al. (2011), which was later revised by Berent (2023). In each vignette, participants must distinguish between one actor’s true belief, and that actor’s awareness of another actor’s different (false) belief (see Appendix 3 for a complete list of items).

##### 2.1.2.6 Demographics

To complete the survey, all participants responded to a demographics questionnaire followed by debriefing.

### 2.2 Results

#### 2.2.1 Attention checks

27% of participants failed the attention check regarding the story’s outcome (e.g., “Did Grace poison her friend?”) and were therefore excluded from analyses. We also checked whether participants correctly attributed actors *intentions*; we hypothesized that participants assigned to the teleological priming group would be more likely to fail the intentionality check in scenarios where intentions and outcomes were mismatched, because they might be more likely to attribute intentions to outcomes. This is not what we found. An independent samples t-test revealed no influence of priming group on intentionality check responses, *t* (213) = −0.42, *p* = 0.676. In other words, teleological priming did not increase participants’ tendency to misattribute intentions based on outcomes.

#### 2.2.2 Endorsement of teleological misconceptions

**Figure 1:**
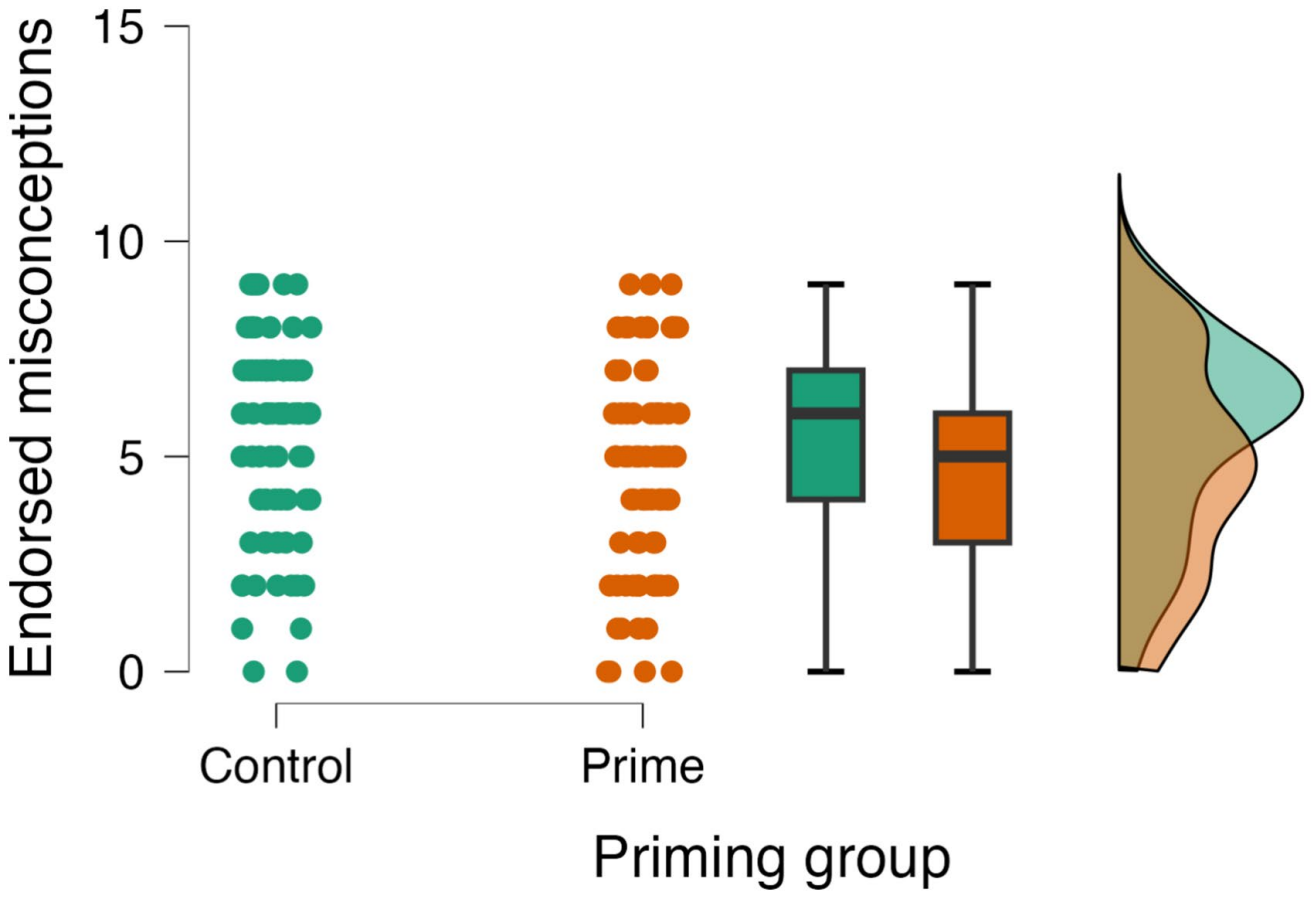
Effects of teleological priming on teleological misconception endorsement.

**Figure 2:**
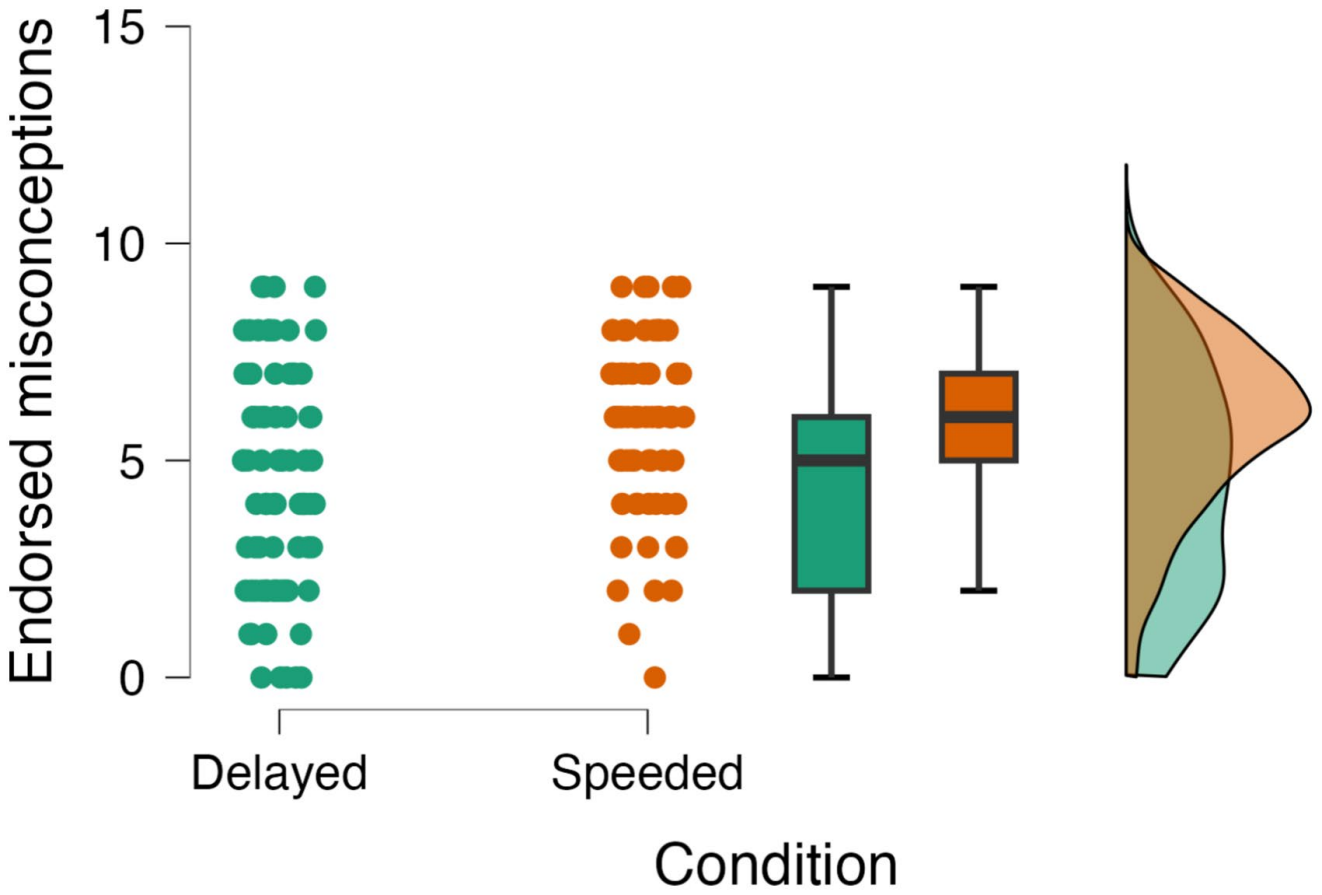
Effects of time pressure on teleological misconception endorsement.

Teleological misconception test sentences that were rated *true* received a score of “1,” and those that were rated *false* received a score of “0.” For each participant, we calculated the total number of test sentences endorsed out of 9. These scores were used to determine the effects of priming group and time pressure on teleological misconception endorsement. We predicted that participants who received the teleology prime, and those who were in the speeded condition, would endorse more teleological misconceptions than participants in the control group or delayed condition. A 2(Prime: Teleology/Control) X 2(Condition: Speeded/Delayed) analysis of variance (ANOVA) revealed a significant effect of priming condition, *F* (1, 153) = 8.09, *p* = 0.005, η_p_^2^ = 0.050. However, rather than *induce* teleological thinking, the teleology prime actually *reduced* it: participants who received the prime endorsed fewer teleological misconceptions (*M* = 4.66, SD = 2.48) than those who received the control reading (*M* = 5.55, SD = 2.24) (see Figure 1). As expected, time pressure significantly impacted teleological misconception endorsement. We found that participants assigned to the speeded condition endorsed more teleological misconceptions (*M* = 5.86, SD = 1.99) than those assigned to the delayed condition (*M* = 4.52, SD = 2.53), *F* (1, 153) = 15.08, *p* < 0.001, η_p_^2^ = 0.090, replicating the literature (Figure 2). The interaction was not significant, *F* (1, 153) = 0.95, *p* = 0.332, η_p_^2^ = 0.006.

#### 2.2.3 Effects of teleology priming on moral judgments

Each participant evaluated two scenarios in which outcomes and intentions were misaligned. For each, participants made a binary punishment judgment and a Likert-style permissibility judgment, totalling four opportunities to make an intent-driven or outcome-driven judgment per participant. We coded responses as either intent-driven or outcome-driven based on the corresponding scenario the participant read (i.e., attempted or accidental). Permissibility judgments were binarized so they could be categorized in this manner. This allowed us to sum the total number of outcome-driven judgments per participant collapsed across story contexts. Because ratings of deserved punishment and ratings of permissibility have been evidenced to involve different influences— the same person may rate something as forbidden, but undeserving of punishment if they believe in prison reform, for instance— we ran two separate analyses (e.g., Cushman, 2008).

##### 2.2.3.1 Permissibility judgments

**Figure 3:**
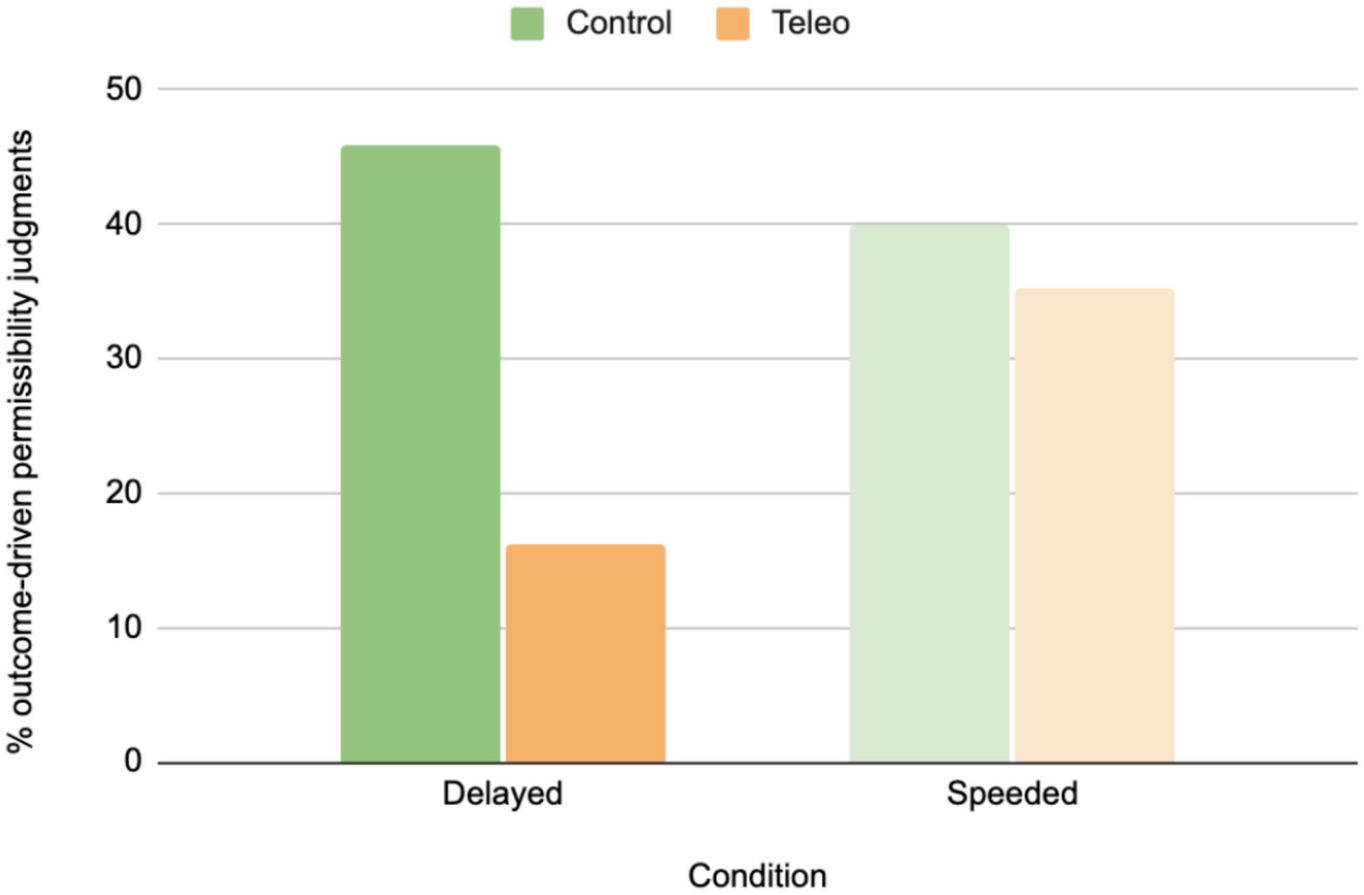
Percentage of permissibility judgements that were outcome-based after teleological and control priming, in the delayed and speeded conditions.

Permissibility judgments were scored as outcome-based when participants judged accidental harm as impermissible or attempted harm actors as permissible. Because outcome-based permissibility judgments were relatively rare (produced by 33% of participants across all relevant trials), participants were binned into two groups: those who, across trials, ever produced such a judgment, and those who did or not. We then conducted 2 (Prime: Teleology, Control) x 2 (Outcome-based judgment: Present, Absent) Chi Square analyses separately for participants in the speeded and delayed conditions. Results showed that outcome-based responses were significantly more frequent in the control condition than in the teleology prime condition when participants were delayed, [*X^2^* (1, 85) = 8.30, *p* = 0.004], but their frequency did not differ in the speeded condition, [*X^2^* (1, 72) = 0.18, *p* = 0.670; see Figure 3].

##### 2.2.3.2 Punishment judgments

**Figure 4:**
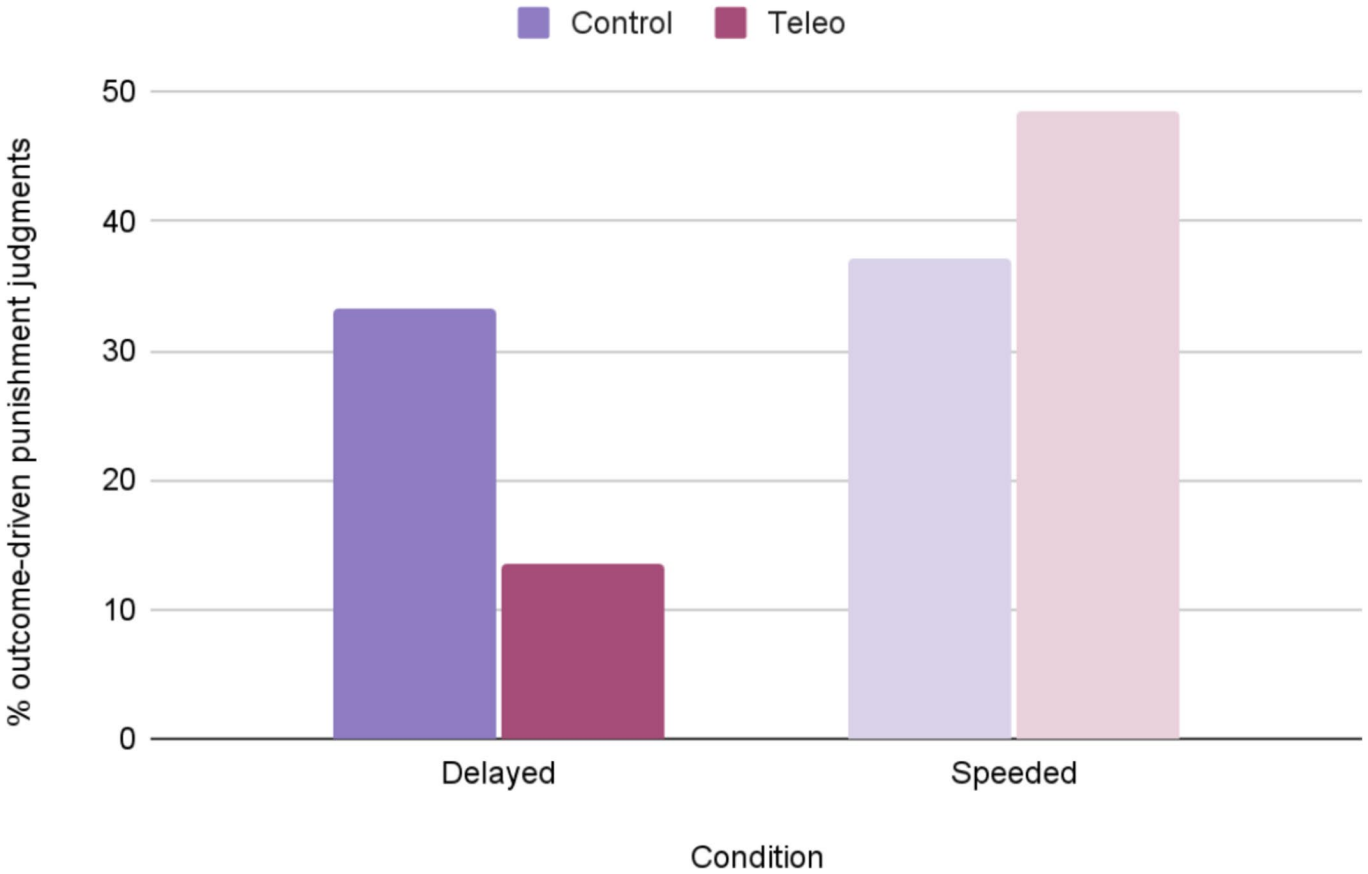
Percentage of punishment judgements that were outcome-based after teleological and control priming, in the delayed and speeded conditions.

We counted a punishment judgment as outcome-based when a participant endorsed punishment for accidental harm or withheld punishment for attempted harm. Again, because outcome-based punishment judgments were relatively rare (produced by 35% of participants across all relevant trials), participants were binned into two groups: those who, across trials, ever produced such a judgment, and those who did or not. We then conducted 2 (Prime: Teleology, Control) x 2 (Outcome-based judgment: Present, Absent) Chi Square analyses separately for participants in the speeded and delayed conditions. Results showed that, again, outcome-based responses were significantly more frequent in the control condition than in the teleology prime condition when participants were delayed, [*X^2^* (1, 85) = 4.41, *p* = 0.036], but their frequency did not differ in the speeded condition, [*X^2^* (1, 72) = 0.97, *p* = 0.324; see Figure 4].

#### 2.2.4 Associations between theory of mind, teleological endorsement, and moral judgments

The number of correct responses to false belief task items were summed for each participant (possible range: 0 to 20). Pearson correlation coefficients were computed to assess the linear relationship between Theory of Mind (ToM) performance, teleological misconception endorsement, and total outcome-driven ratings of punishment and permissibility. No relationships were significant. There was no correlation between ToM performance and teleological misconception endorsement, outcome-driven punishment judgments, nor outcome-driven permissibility judgments. Additionally, we found no correlations between teleological misconception endorsement and moral ratings of punishment and permissibility, though both judgments of morality were strongly correlated (see Table 1). Limited by binary response formats, we worried that our variables were unable to capture sufficient variability to detect a correlation between teleology and moral reasoning. We address this concern in Study 2.

**Table 1:** Pearson correlation coefficients between theory of mind task, teleology endorsement task, outcome-based punishment ratings, and outcome-based permissibility ratings.

Variable	Outcome-driven punishment	Outcome-driven permissibility	Teleological misconception endorsement
ToM	−0.08	−0.02	0.08
Outcome-driven punishment judgments	–	0.34***	0.12
Outcome-driven permissibility judgments	–	–	0.09

*p* < 0.05**; *p* < 0.01; *p* < 0.001***.

### 2.3 Discussion

In Study 1 we sought to investigate if teleological reasoning influences adults’ moral judgments, and we hypothesized that if we primed teleological reasoning, adults would endorse more teleological misconceptions and make more outcome-driven moral judgments. Our prime was unsuccessful at inducing teleological thinking. However, the manipulation did affect teleology in the opposite direction, with participants who received the teleology prime endorsing fewer teleological misconceptions than those in the control group. We speculate that by drawing explicit attention to teleological reasoning, our intervention served as a refutation text, inadvertently increasing students’ metacognitive monitoring of their own teleological tendencies, thereby rendering them less likely to endorse teleological misconceptions (see Pickett et al., 2022). Of central importance, however, is that our manipulation also impacted moral judgments in a similar pattern. While we predicted that the teleology prime would *facilitate* teleological thinking, thus leading to *more* outcome-driven moral judgments, it actually *suppressed* teleological thinking and led to *fewer* outcome-driven moral judgments.

According to this reasoning, we should have seen that participants assigned to the priming group were less likely to fail an intentionality check. Notably, all participants in both the control and priming groups responded to the attention check and intentionality check in their own time, without time pressure. We would therefore expect participants who received the teleology prime, which has evidently acted as a refutation prime, to reason correctly about the intentions of the actor. However, the fact that participants attributed intention correctly at the same rate regardless of priming group seems to contradict the teleological account, and instead suggests that there may be some other reason, unrelated to intention, that explains apparently outcome-based judgements.

It is possible, for example, that participants inferred negligence, rather than intention, from the negative outcomes they observed. This kind of reasoning could lead to judgments that appear to be outcome-based but are actually influenced by perceptions of carelessness or irresponsibility on the part of the actor (Kneer and Skoczen, 2023; Margoni et al., 2019; Nobes and Martin, 2022; Nobes et al., 2023). Such perceptions may be derivative of hindsight bias, whereby negative consequences precipitate retrospective blame (Kneer and Skoczen, 2023; Margoni et al., 2019). The results of the intentionality check alongside these explanations suggest that outcome-based judgments may stem from cognitive biases that operate independently of the actor’s perceived intentions.

Study 1 also replicated findings from time pressure studies which suggest that under time pressure, adults are more likely to revert to their intuitions of promiscuous teleology (Roberts et al., 2020; Kelemen et al., 2013; Kelemen and Rosset, 2009; Steiner et al., 2016). Specifically, participants in the speeded group endorsed more teleological misconceptions than those in the delayed group.

Notably, for both permissibility judgments and punishment judgments, participants made the least outcome-based judgments when they were in a delayed condition and exposed to teleological refutation text. This would align with evidence from Steiner et al. (2016), who found that between participants with pre-existing intentional agent beliefs and atheists, the difference in acceptance of teleology was in the unspeeded condition, when everyone had time to correct their intuitions. For this reason, we designed Study 2 with unspeeded conditions only, so as to capture the greatest effect of manipulating the proposed relationship.

The present findings also inadvertently add to existing research showing the efficacy of refutation texts— readings designed for conceptual change by correcting and improving sense-making of counterintuitive scientific notions— as an intervention tool. For example, replacing standard expository text with refutation text that explicitly mentions common misconceptions, refutes them, and contrasts them with correct, scientific explanations has been evidenced to significantly improve undergraduate students’ conceptual understanding of scientific content such as biological evolution (Asterhan and Resnick, 2020) and antibiotic resistance (Pickett et al., 2022). Such advantageous effects have been shown to be both immediate and enduring (Asterhan and Resnick, 2020) as well as unconstrained by scientific domain; one review of 31 studies concerned with a range of scientific topics concluded that refutation text, rather than traditional expository text, is more likely to result in conceptual change (Tippett, 2010). We believe the unintended consequences of our teleology prime were those of a refutation text, which provided participants with the tools to overcome their intuitive misconceptions and correct them when given sufficient time.

In sum, Study 1 provided some evidence connecting teleological reasoning with moral judgments; reducing teleological reasoning in turn reduced outcome-based moral judgments. Study 1 also provided evidence against such a relationship; teleology and outcome-based moral judgment were uncorrelated, and our manipulation did not affect perceived intention in the way it did moral judgments. To further investigate this relationship, we sought to develop a more implicit methodology for priming teleological reasoning. If inducing teleological thinking with an implicit prime leads participants to endorse more teleological explanations and make more outcome-based moral judgments that misinterpret intention, it would provide evidence for our hypothesis that people possess the implicit assumption that all consequences are the result of aligned intentions.

## 3 Study 2

In Study 1, we saw that outcome-based moral judgments were reduced after a teleology refutation prime. The aim of Study 2 was to design a teleology prime that did not function as a refutation. Specifically, we wanted the prime to be more implicit so as to subliminally encourage teleological reasoning. Should participants who receive an implicit teleology prime make more outcome-driven moral judgments than those who did not, we could draw the conclusion that the frameworks are implicitly related.

To achieve this, we manipulated the order of task presentation so that participants were randomly assigned to a condition in which they either received the teleology endorsement task first (“Teleo → Moral”) or the moral judgment task first (“Moral → Teleo”). We hypothesized that participants who read and responded to teleological statements before they read and responded to scenarios concerned with human intentions and outcomes would be primed to make more outcome-driven moral judgments under the teleological assumption that outcomes indicate intentions. Otherwise, Study 2 was nearly identical to Study 1 with a few amendments. We removed the explicit priming tasks from Study 1, as well as the Theory of Mind task given its nonrelevance. We chose not to manipulate time pressure as in Study 1, seeking to investigate the nature of responses when participants were allowed time to reason about their responses but were not provided tools to override intuitions. We also increased the possible variability of responses to moral judgment questions and teleological endorsement by putting all responses in a Likert scale format, hoping for more sensitive measures of individual differences.

In addition to H1, we explore the following hypothesis:

*H3*: If teleological reasoning predicts adults’ moral judgments, then individuals with stronger teleological tendencies will be more likely to make outcome-driven moral judgments.

### 3.1 Method

#### 3.1.1 Participants

The participants in Study 2 were 208 adults recruited via Prolific, 74 of which were excluded for failing a relevant attention check. Of the 134 participants included in the analysis, 72 identified as women, 59 as men, 1 as nonbinary, and 2 declined to identify. Racial demographic information was provided by 100% of participants. Of those, 74% identified as white, 9% identified as multiracial, 6% identified as Hispanic/Latinx, 5% identified as Middle Eastern, 3% identified as East Asian, 1.5% identified as Black, and 1.5% identified as other. Respondents were paid at a rate of $11.49 per hour, prorated to our 8 min task, and participation was restricted to workers in the United States.

#### 3.1.2 Materials and procedure

The procedure of Study 2 is similar to that of Study 1 but without an explicit priming task, Theory of Mind task, or time constraints. Instead, we manipulated task order as our independent variable: Participants were randomly assigned to either a Teleo → Moral condition or a Moral → Teleo condition. The Teleology Endorsement task was identical to the one used in Study 1 except for the response format. We changed the binary (True/False) response format to a Likert scale in Study 2 (“Strong disagree”; “Disagree”; “Somewhat disagree”; “Somewhat agree”; “Agree”; “Strongly agree”) to increase response variability. Similarly, the Moral Judgment task in Study 2 mirrored that of Study 1 but with expanded scales for punishment (“Do you think Grace should be punished?”; [“No, not at all”; “Only a little”; “Yes, moderately”; “Yes, severely”]) and permissibility (“Grace putting the substance in was:”; [“Absolutely unacceptable”; “Unacceptable”; “Somewhat unacceptable”; “Somewhat acceptable”; “Acceptable”; “Absolutely acceptable”]) judgments. Randomization of the Moral Judgment task was designed so that each participant saw one scenario of attempted harm and one scenario of accidental harm, counterbalanced between story contexts. Because we were only concerned with scenarios in which actors’ intentions and the outcomes that occurred were misaligned, we did not include scenarios of intentional harm or neutral (non-intentional, non-outcome) events. Thus, participants made judgments (one punishment, one permissibility) about two total scenarios.

### 3.2 Results

#### 3.2.1 Attention checks

36% of participants failed an attention check regarding the outcomes caused by the actor and were therefore excluded from analyses. Like in Study 1, those who failed only an intentionality check remained included. Of the remaining participants, none failed the intentionality check following the Jellyfish story in either the attempted or accidental contexts. In contrast 26% of participants failed the intentionality check following the Coffee story in the attempted harm context, whereas no one in the accidental did so. This difference was significant (X^2^(1,134) = 16.3, *p* < 0.001), and suggests that the attempted harm version of the Coffee story may have been confusing for participants.

#### 3.2.2 Effects of task order on moral judgments and teleological misconceptions

If responding to teleological probes first primed teleological thinking, which in turn increased outcome-based moral permissibility and punishment judgements, we would expect differences based on task order (i.e., teleology first versus morality first). Specifically, we would expect teleology-first participants to rate attempted harm scenarios as more permissible and less deserving of punishment than morality-first participants (due to lack of a negative outcome), and likewise, that teleology-first participants would rate accidental harm scenarios as less permissible and more deserving of punishment (due to negative outcome) than morality-first participants.

**Figure 5:**
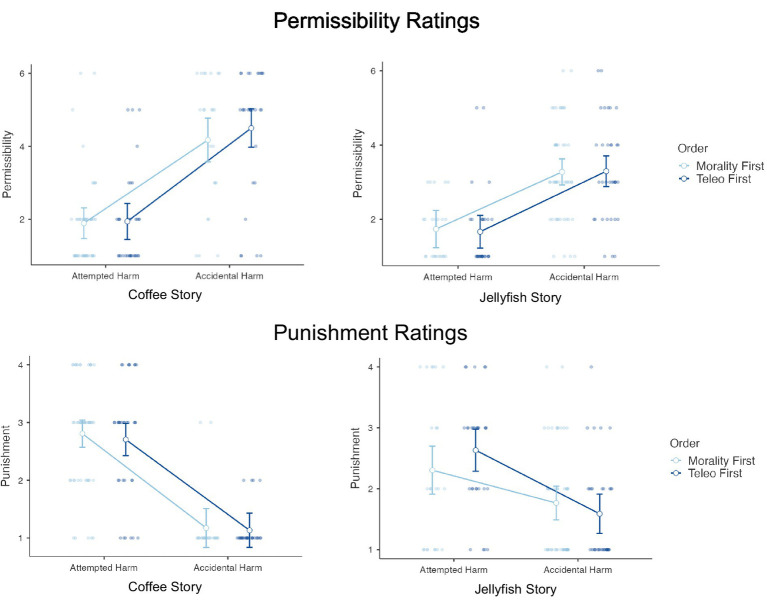
Order effects on moral judgments of accidental and attempted harm: 2 (Scenario type: Attempted harm, Accidental harm) x 2 (Order: Morality first, Teleology first) ANOVAs on permissibility ratings and punishment ratings for the Coffee and Jellyfish scenarios.

To test these predictions, we conducted separate 2 (Scenario type: Attempted harm, Accidental harm) x 2 (Order: Morality first, Teleology first) ANOVAs on permissibility ratings and punishment ratings for the Coffee and Jellyfish scenarios. Results are depicted in Figure 5. For both scenarios, attempted harm was rated less permissible than accidental harm (Coffee Scenario: *F* (1,130) = 86.77, *p* < 0.001, η^2^ = 0.399; Jellyfish Scenario: *F* (1,130) = 53.31, *p* < 0.001, η^2^ = 0.291). Presenting teleological items first had no effect on permissibility ratings (Coffee Scenario: *F* (1,130) = 0.52, *p* = 0.473; Jellyfish Scenario: *F* (1,130) = 0.02, *p* = 0.899). We observed the same pattern for punishment ratings: attempted harm was considered more worthy of punishment than accidental harm (Coffee Scenario: *F* (1,130) = 120.40, *p* < 0.001, η^2^ = 0.480; Jellyfish Scenario: *F* (1,130) = 21.58, *p* < 0.001, η^2^ = 0.140). Presenting teleological items first had no effect on punishment ratings (Coffee Scenario: *F* (1,130) = 0.24, *p* = 0.625; Jellyfish Scenario: *F* (1,130) = 0.20, *p* = 0.658). In sum, we find no evidence that responding to teleological items first, and presumably rendering teleological reasoning salient, had any effect on permissibility or punishment judgments in these scenarios.

#### 3.2.3 Associations between teleology and moral judgments

**Figure 6:**
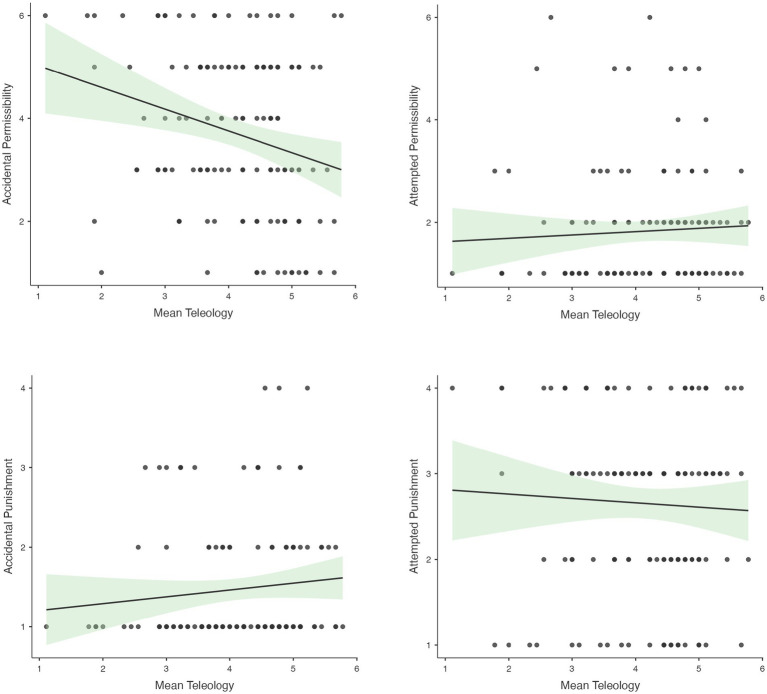
Individual teleology and moral judgments: Correlations between teleology endorsement ratings and permissibility and punishment judgments in the context of accidental and attempted harm scenarios.

Although there were no effects of task order, which we considered to be an implicit teleology prime, we were still interested to see if individual differences in teleological tendencies predicted moral judgments. To do so we examined correlations between teleology ratings and permissibility and punishment judgments in the context of accidental and attempted harm scenarios, respectively. If individual differences in teleological reasoning were associated with increased outcome-based judgments, we would expect a negative correlation between teleology and permissibility in the accidental harm context, and a positive correlation in the attempted harm context (i.e., higher teleological reasoning should be associated with increased consideration of outcome, which is negative for accidental harm scenarios but not for attempted harm scenarios). The same rationale would predict a negative correlation between teleology and punishment in the attempted harm context, and a positive correlation in the accidental harm context. Results are depicted in Figure 6. We observed the predicted correlation for permissibility judgments in context of accidental harm, *r* (131) = −0.252, *p* = 0.003; in this context, participants with higher teleological reasoning scores tended to judge accidental harm as less permissible across coffee and jellyfish scenarios. Correlations in the other conditions ranged from −0.047 to 0.140 and did not approach significance (*p* > 0.234).

### 3.3 Discussion

In Study 2 we attempted a more subtle manipulation of teleological reasoning by presenting the teleological prompts either before (to prime teleological reasoning) or after the morality questions. We found no evidence that this manipulation had any effect on moral judgements of permissibility or punishment in either story.

We did find limited support for the hypothesis that individuals with stronger teleological tendencies are more likely to make outcome-driven moral judgments. However, these findings were highly context-dependent, and evidence only when participants made permissibility judgments in the accidental harm context. In other words, those who were more teleological were more likely to consider the actor morally wrong in a context of a negative outcome, even though it was an accident.

While this finding was expected and aligns well with our theory, it was not found in any attempted harm story contexts, nor among any ratings of punishment. This is likely because negative events elicit an increased search for causal explanations (as opposed to a negative non-event, such as attempted harm) (Waytz et al., 2010; Taylor, 1991). This fits with the finding that entities that produce negative outcomes seem more intentional than those that produce positive ones (Morewedge, 2009) and that people attribute more intentionality to those who commit evil deeds than good deeds (Knobe, 2006). It is not surprising, then, that the accidental harm scenario was the scenario most closely related to teleological explanation. Previous work in moral judgment and development supports the idea that negative outcomes, particularly those involving harm, are more likely to prompt attributions of intent and moral condemnation (Buon et al., 2016; Cushman et al., 2013; Martin et al., 2021a,b). If positive or neutral outcomes do not elicit this same response, we would not expect to see the same relationship between teleology and moral evaluation in attempted harm scenarios.

Finally, the observed relationship between teleology and moral judgments was also specific to moral ratings of permissibility, as we did not see a significant relationship between teleology and punishment ratings. This finding replicates literature which suggests that moral evaluation of permissibility and moral evaluation of punishment are derived from distinct processes (Cushman, 2008). For example, cognitive load has been shown to have a greater effect on judgments of moral wrongness than judgments of deserved punishment (Martin et al., 2021a,b). Judgments of permissibility have furthermore been found to rely principally on the intentions of an agent, while judgments of punishment incorporate a broader range of factors, including causal responsibility and severity of outcome (Cushman, 2008, 2013). Therefore, permissibility ratings should be more sensitive to teleological manipulation or tendencies that diminish the role of intentions, whereas punishment judgments are less influenced by such factors due to their reliance on outcome-based reasoning.

In sum, although we found no evidence in Study 2 that engaging in teleological reasoning had any impact on subsequent moral judgment, we did find limited support for a relationship between individual teleological tendencies and moral reasoning. While our findings were highly context-dependent, they nonetheless provide some evidence that a teleological bias may contribute to at least some moral judgments.

## 4 General discussion

Young children endorse more teleological explanations for biological, behavioral, and nonliving kind properties than do adults (Kelemen, 1999a,b; Coley et al., 2017), but the same effect is observed when adults’, including experts’, cognitive resources are incapacitated (Kelemen et al., 2013). Neither age nor expertise are sufficient to replace the teleological bias, suggesting teleology is a persistent, domain-general, cognitive default that emerges in response to novel, complex stimuli or in situations where overriding executive function is unavailable. The same may be true for moral reasoning: while young children seem to make more outcome-based moral judgements than adults do, adults have still been observed to resort to similarly patterned judgments under cognitive load (Buon et al., 2013a,b). At the outset of this work, we theorized that a cognitive default by which outcomes are assumed to be intentional could explain this pattern of observations in children and adults with limited cognitive resources alike.

Across two studies, we investigated the teleological bias and its implications for moral judgment. In Study 1, we found that participants who received an explicit teleology prime—which we suppose unintentionally induced refutation—endorsed fewer teleological misconceptions and made fewer outcome-driven moral judgments. We also found that participants were more likely to endorse teleological misconceptions under speeded conditions, replicating the literature (Roberts et al., 2020; Kelemen et al., 2013; Kelemen and Rosset, 2009; Steiner et al., 2016). The main effect of priming on moral judgments in the delayed condition but not the speeded condition could suggest that, when given sufficient time to override their intuitions, participants evoked the new reasoning patterns provided by the refutation text.

Study 2 provided some evidence of an implicit relationship between teleological thinking and moral reasoning. While our “prime,” which depended on task order, did not engender group differences, we did reveal that individual differences in teleological tendencies were related to moral judgments in certain contexts. Specifically, participants who were more teleological were more likely to make outcome-based moral judgments of permissibility when evaluating an accidental harm scenario. According to the proposed theory, this would suggest that when evaluating accidental harm, participants with a strong teleological bias reason that the negative outcome can be explained by purposeful intentions, resulting in a judgment that condemns the actor for the harm they caused even though it was accidental. However, the results of the intentionality checks, which revealed that many participants correctly recognized an actor’s intentions despite making outcome-based judgments, evidence the contrary. This pattern undermines the claim of a cognitive default that invariably assumes all outcomes are intended.

One interesting finding from our study was that the relationship between teleological bias and moral judgment was specific to moral ratings of permissibility in accidental harm scenarios. Participants who were more teleological were more likely to judge accidental harm as morally wrong. They were not, however, less likely to condemn attempted (but failed) harm, nor were their judgments of deserved punishment affected by their teleological bias. Interestingly, the study by Martin et al. (2021a,b) also demonstrated the selective manipulability of permissibility judgments of accidental harm, finding a differential impact of cognitive load on attempted and accidental harm, as well as depending on the type of moral probe.

This evidence aligns with the broader literature suggesting that judgments of permissibility are systematically different from judgments of punishment. For example, Cushman’s (2008) and (2013) dual-process model posits that permissibility judgments are primarily based on an agent’s intentions, whereas punishment judgments are influenced by both intention and the causal connection between the agent and harmful consequence. This distinction can explain why two drunk drivers might be rated as equally wrong or impermissible, but one might be deemed more deserving of punishment if they unintentionally cause injury to a third party—a phenomenon otherwise known as moral luck (Nagel, 1979; Williams, 1981; Martin and Cushman, 2016). Punishment judgments incorporate not only the agent’s intentions but also their causal role and the severity of the outcome, which may explain why teleological manipulation had a stronger effect on permissibility judgments. Future research should explore these distinctions further by examining how teleological priming affects different types of moral judgments.

Our work across these two studies seeks to investigate the possibility that the teleological bias underpins the way humans make moral judgements. Of core importance is that we were able to manipulate moral judgments by suppressing teleology, and that in specific contexts, we evidenced that adults who are more teleological are also more likely to make apparently outcome-based judgments. However, the lack of any effect of our second order manipulation and the unchanged rate of accurate intention attributions in both studies directly challenge the central premise of our teleological bias account. Even when associations between teleological reasoning and moral judgments were found, they could be explained by many other factors, like attention, intelligence, arousal, or need for cognition. The apparent absence of a role for perceived intentionality – which would be required if the teleological hypothesis were correct – suggests that this kind of alternative explanation is more likely.

This work operated under a definition of teleology characterized by intention overattribution: a perspective in which any observed outcome is implicitly assumed to have been intended by some agent. Our studies aimed to test whether this “teleological default” could explain why people sometimes seem to focus on outcomes (e.g., a harmful consequence) over explicit intention when making moral judgments. Despite setting out with this intention-centered framework, the data revealed a more nuanced relationship between teleological reasoning and moral evaluation, ultimately indicating that teleology need not always hinge on agentic intent—even when it exerts an influence on moral judgments.

Historically, many discussions of teleology have conflated it with overt attributions of intention (Dennett, 1987). However, the evidence presented here suggests that even when adults correctly identify an action as accidental, they may still judge the action’s outcome in a manner consistent with a teleological “purpose-based” mindset. For instance, under time pressure, participants reverted to teleological explanations and made more outcome-driven moral judgments—even while correctly recognizing that the actor did not intend harm. These findings align with Scott’s (2022) argument that teleology need not rely on supernatural or agent-driven intention: people can interpret events as if they serve a purpose—whether cosmic, functional, or otherwise—without invariably concluding that any specific actor orchestrated them. Critically, participants’ accurate grasp of intentions suggests that teleology and intentionality are not one and the same. Rather, teleological bias can coexist with correct attributions of an agent’s goals, subtly framing outcomes as less random, more “destined.” In turn, people may place heightened emphasis on the outcome of an event, perceiving it as non-accidental or as part of a purposeful framework—even if they explicitly know the agent was unaware or did not intend harm.

The heightened significance of bad outcomes under a teleological mindset may help clarify why it’s associated with permissibility judgments but not punishment judgments. According to Cushman’s (2008) and (2013) dual-process model, punishment decisions often rely on the actual outcome regardless of intent—so *accidental harm* already carries negative consequences that make punishment plausible. *Permissibility* judgments, by contrast, are presumed to hinge more strongly on the actor’s mental states. In our initial view—where teleology was taken to mean overattributing intention—we assumed that teleologically inclined participants who harshly judged accidental harms did so because they believed these harms were *actually intended*. However, our data show that participants still recognized an action as accidental, suggesting that teleology need not imply intention. Rather, it may amplify the *salience* or *seriousness* of the outcome itself, especially if that outcome is a purpose violation (Lewry et al., 2023). Consequently, it is the *outcome-based* element of teleology that drives participants to see the accidental harm as morally wrong—even though they know the actor did not intend it. Within accidental harm scenarios, then, the question of *whether something is permissible* becomes vulnerable to a “purpose-laden” bias, whereas punishment judgments—already determined largely by the resulting harm—remain comparatively stable.

Beyond a more nuanced understanding of teleology, other, less speculative explanations are likely at play. Participants might have inferred negligence (Nobes and Martin, 2022; Nobes et al., 2023) in the case of the mislabeled white powder in the Coffee story, with hindsight bias (Kneer and Machery, 2019; Kneer and Skoczen, 2023; Margoni et al., 2019, 2023) potentially inducing ex post facto judgments of fault for the accident. Outcome bias (Baron and Hershey, 1988) and motivated blame, which posits that personal biases or motivations may influence how blame for bad outcomes is assigned, often leading to disproportionate condemnation (Alicke, 2000), could also explain our findings. These alternative accounts offer compelling explanations for why participants may have made outcome-based judgments independent of their teleological tendencies.

Our findings should therefore be interpreted within the context of alternative explanations. In future research, it would be informative to explicitly test how different biases interact with teleological reasoning to shape moral judgments. For example, Nobes et al. found that perceived negligence explained more of the outcome effect than perceived intention, suggesting that the teleological bias may interact with other factors rather than operate in isolation (2023).

## 5 Conclusion

We tested the hypothesis that inappropriately outcome-based moral reasoning could be attributed to disinhibition of teleological thinking, leading to a bias by which outcomes are assumed to be intentional, regardless of actual intent. Our findings reveal mixed evidence for this hypothesis. While our findings suggest that teleological bias might play some part in shaping certain moral judgments, these effects were small and inconsistent. Indeed, the null findings on intentionality checks point to alternative explanations—like outcome bias and negligence—that may better explain outcome-based moral judgments.

Taken together, these findings suggest that the teleological “default” is not equivalent to a blanket assumption that outcomes are always intended. Instead, teleology may often manifest as a broader impression that outcomes themselves are *non-accidental* or purposeful in some overarching framework. Under certain conditions—like time pressure or absence of teleology refutation—this mindset may tilt moral judgments in an outcome-based direction, particularly when participants evaluate whether accidental harm is “morally permissible.” Yet, as shown by the consistently accurate recognition of *accidental* versus *intentional* action, teleological reasoning and intention detection are dissociable processes. By distinguishing functional or purpose-focused teleology from explicit attributions of intention, we can better understand why people may intuitively treat outcomes as “meaningful” without necessarily perceiving every outcome as an intended act.

## Data Availability

The raw data supporting the conclusions of this article will be made available by the authors, without undue reservation.
