# Supplementary material for: Means to an end: teleological bias in moral reasoning
Source: Front Psychol. 2025 Jun 27;16:1380048. doi: 10.3389/fpsyg.2025.1380048 (PMC12245872; doi:10.3389/fpsyg.2025.1380048)
Supplement: Supplementary file 1 [file Data_Sheet_1.PDF]

Appendix

Teleology Prime	Control
Please carefully read the following text. Take your time to fully comprehend the content of the passage.<br><br>From a young age, people try to make sense of the world around them in causal terms. Humans like to ask "Why?" and fall back on default cognitive tools when the answers are unclear. One such cognitive tool is teleological explanation, which assumes that objects or events occur for a purpose.<br><br>Teleological explanations are a central aspect of adult thought, leading us to think about entities in terms of their intended function. For example, they constrain our thinking about human-made artifacts. It is teleology that helps us to reason that clocks were created to tell time. Teleology also plays an important part in our reasoning about living things, leading us to assume, for example, that biological structures such as eyes and hearts are designed for a purpose.<br><br>For most scientifically educated adults, however, teleological explanations tend to apply no further than this. A nonliving natural entity such as a river may be capable of performing any number of activities—making fields fertile, providing a habitat for fish—but these activities are consequences of the mechanical forces that caused it to form, rather than explanations for why it formed. In other words, for most adults with a knowledge of natural mechanisms, a pointy part on an animal might exist to perform some protective function, but a pointy part on a rock is the purposeless result of a physical process such as erosion. Importantly, without formal knowledge, reliance on teleological explanations may cause scientific misconceptions, leading to incorrect assumptions like "snowflakes fall to cool the Earth." To a significant extent, then, Western adults' teleological reasoning is demarcated along expert lines.	Please carefully read the following text. Take your time to fully comprehend the content of the passage.<br><br>Reviving the practice of using elements of popular music in classical composition, an approach that had been in hibernation in the United States during the 1960s, composer Philip Glass (born 1937) embraced the ethos of popular music in his compositions. Glass's work has been associated with minimalism, being built up from repetitive phrases and shifting layers. Glass describes himself as a composer of "music with repetitive structures", which he has helped evolve stylistically. In late 1970s Berlin, Glass composed two symphonies based on music by rock musicians David Bowie and Brian Eno, but the symphonies' sound is distinctively his. Glass's uniquely styled compositions have won over audiences in the opera house, the concert hall, the dance world, in film, and in popular music. Popular elements do not appear out of place in Glass's classical music, which from its early days has shared certain harmonies and rhythms with rock music. Yet this use of popular elements has not made Glass a composer of popular music. His music is not a version of popular music packaged to attract classical listeners; it is high art for listeners steeped in rock rather than the classics.

Please come up with an explicit teleological explanation for each of the following prompts. For example, if a prompt asked, "Why do giraffes have long necks?" you might respond, "Giraffes have long necks to reach leaves on high branches." Your responses can be scientifically inaccurate or logically unsound as long as they are teleological.<br><br>Please respond in complete sentences.<br><br>*Why do candles have wicks?*<br><br>*Why are ears of corn wrapped in husks?*<br><br>*Why do humans make art?*	1. Select only one answer choice.<br><br>The passage addresses which of the following issues related to Glass's use of popular elements in his classical compositions?<br><br>● How it is regarded by listeners who prefer rock to the classics<br>● How it has affected the commercial success of Glass's music<br>● Whether it has contributed to a revival of interest among other composers in using popular elements in their compositions<br>● Whether it has had a detrimental effect on Glass's reputation as a composer of classical music<br>● Whether it has caused certain of Glass's works to be derivative in quality<br><br>2. Consider each of the three choices separately and select all that apply.<br><br>The passage suggests that Glass's work displays which of the following qualities?<br><br>☐ A return to the use of popular music in classical compositions<br>☐ An attempt to elevate rock music to an artistic status more closely approximating that of classical music<br>☐ A long-standing tendency to incorporate elements from two apparently disparate musical styles<br><br>3. Select the sentence that distinguishes two ways of integrating rock and classical music.<br><br>(1) Reviving the practice of using elements of popular music in classical composition, an approach that had been in hibernation in the United States during the 1960s, composer Philip Glass (born 1937) embraced the ethos of popular music in his compositions. (2) Glass's work has been associated with minimalism, being built up from repetitive phrases and shifting layers. (3) Glass describes himself as a composer of "music with repetitive structures", which he has helped evolve stylistically. (4) In late 1970s Berlin, Glass

	composed two symphonies based on music by rock musicians David Bowie and Brian Eno, but the symphonies' sound is distinctively his. (5) Glass's uniquely styled compositions have won over audiences in the opera house, the concert hall, the dance world, in film, and in popular music. (6) Popular elements do not appear out of place in Glass's classical music, which from its early days has shared certain harmonies and rhythms with rock music. (7) Yet this use of popular elements has not made Glass a composer of popular music. (8) His music is not a version of popular music packaged to attract classical listeners; it is high art for listeners steeped in rock rather than the classics.

Appendix 1. Priming & Control Texts and Comprehension Questions

Teleological sentences

Sentence type	Subtype	Item
Test	Nonbiological teleological	Trees produce oxygen so that animals can breathe.
		Rain falls in order to allow plants to grow.
		The Earth has an ozone layer in order to protect it from UV light.
		Water exists so that life can survive on Earth.
		Particles collide in order to produce chemical reactions.
	Biological teleological	Microbes convert nitrogen in order to enrich the soil.
		Germs mutate in order to become drug resistant.
		Lemurs have adapted in order to avoid extinction.
		Parasites multiply in order to infect a host.
Control	False teleological	People put coins into meters in order to get rid of spare change.
		Cows have udders in order to allow farmers to milk them.
		Hair becomes grey so that people can look older.
		Musicians have two hands in order to play instruments.
		Kittens have soft fur so that people will want to pet them.
		People chew food in order to strengthen their jaw muscles.
		Window blinds have slats so that they can capture dust.
		Houses have doorbells in order to make dogs bark.
		Mice run away from cats in order to get exercise.
		Lamps shine brightly so that they can produce heat.
	True teleological	Alarm clocks beep in order to wake people up.
		Bicycles have handlebars so that people can steer them.
		People wear contact lenses in order to see more clearly.
		Doctors prescribe antibiotics in order to treat infections.
		Children wear mittens in the winter in order to keep their hands warm.
		People buy microwaves in order to heat their food.
		Pencils exist so that people can write with them.
		Women put on perfume in order to smell pleasant.
		Schools exist in order to help people learn new things.
		People have chairs so that there is a place to sit.
		Stoplights change color in order to control traffic.

Appendix 2. Teleological statements included in the Teleology Endorsement Task by category.

#	Story	Answer
1.	The morning of high school dance Sarah placed her high heel shoes under her dress and then went shopping. That afternoon, her sister borrowed the shoes and later put them under Sarah's bed. *Sarah gets ready assuming her shoes are under the dress.*	yes
2.	John told Mary that he had lost his keys, and the two of them go searching. While John looks outside, Mary finds the keys, but since she's in a hurry, she just places them on the table at the entrance and leaves. *When John returns, he expects to see the keys on the table.*	no
3.	Expecting the game to be suspended because of the rain, the Garcia family took the subway home. The score was tied, 3-3, and on their way home, the family had no internet connection. During their commute the rain stopped and the game soon ended with a score of 5-3. *The Garcia family arrives home believing the score is 5-3.*	no
4.	Susie parked her sports car in the driveway. In the middle of the night, Nathan moved her car into the garage to make room for his minivan. When Susie wakes up, *she expects to see her car in the driveway.*	Yes
5.	When Lisa left Jacob, he was deep asleep on the beach. A few minutes later a wave woke him. Seeing Lisa was gone, Jacob decided to go swimming. *Lisa now believes that Jacob is sleeping.*	Yes
6.	Larry chose a debated topic for his class paper due on Friday. The news on Thursday indicated that the debate had been solved, but Larry never read it. *When Larry writes his paper he thinks the debate has been solved.*	No
7.	A window wiper was commissioned by a CEO to wipe an entire building. He finished the right side, but his platform gets stuck before he could do the left side, so he reports the problem to the CEO. Overnight, the cleaner comes up with a solution, and early in the morning, he diligently returns to finish the job. *When the CEO comes to work at noon, he expects to see all of the windows are cleaned.*	No
8.	Sally and Greg called ahead of time to make a reservation for the back-country cabin. The park ranger forgot to write down the reservation and two other hikers got to the cabin first. *When the hikers arrive, they expect the other hikers to be in their room.*	No
9.	Rather than driving to work, today Amy decided to walk. When George woke up, he saw her car in the drive. Amy's room was quiet and dark. George knows that when Amy is sick, she lies down in a dark room. *George expects Amy is in her room, sick with migraine.*	yes
10.	Laura didn't have time to braid her horse's mane before going to camp. While she was at camp, William brushed Laura's horse and braided the horse's mane for her. *Laura returns assuming that her horse's hair isn't braided.*	Yes
11.	At night a bear broke into a cooler near a tent and drank the soda. Five hours later, the campers woke up and went to their cooler for breakfast. *In the cooler, the campers expect to find soda.*	Yes
12.	Anne made lasagna in the blue dish. After Anne left, Ian came home and ate the lasagna. Then he filled the blue dish with spaghetti and replaced it in the fridge. *Anne thinks the blue dish contains spaghetti.*	no
13.	Jenny put her chocolate away in the cupboard. Then she went outside. Alan moved the chocolate from the cupboard into the fridge. Half an hour later, Jenny	yes

	came back inside. *Jenny expects to find her chocolate in the cupboard.*	
14.	The weather was so warm today that all the tulips in Pam's backyard suddenly bloomed. The tulips next to Pam's office still have not yet flowered, though. Pam has been at work all day. *Driving home after work, Pam supposes her tulips have not bloomed.*	Yes
15.	When the class' science test was handed back, Shannon was mistakenly given Adam's test. A large B was written on the front of Adam's test, but Shannon's actual grade was an A. *Shannon believes she received a B on the exam.*	yes
16.	Every day Jill goes to the coffee shop on the corner and orders a latte, her favorite drink. Today, the cashier misunderstands Jill and prepares a mocha instead. *Jill thinks her drink will taste like a mocha.*	No
17.	Hopeful to catch a prize fish, George went fishing. That afternoon, he saw his fishing line bend over as if he had caught a big fish. Actually, George's fishing pole had snagged a small tire. *At the end of the fishing line, George expects to see a tire.*	No
18.	The girls left ice cream in the freezer before they went to sleep. Overnight, the power to the kitchen was cut and the ice cream melted. *When they get up the girls believe the ice cream is melted.*	No
19.	Ken told Andrea that he was going shopping for sandals. At the shoe store, Ken noticed a very nice pair of boots on sale, and bought them instead. *When he meets Andrea, she believes Ken's shoe store bag contains boots*.	No
20.	Jeff is colorblind, so he cannot tell subtle differences in color. To help him dress up, his housekeeper normally keeps the pink and white shirts in different drawers, but this week, she is sick, and her replacement placed the white shirts at the bottom drawer, where the pink shirts usually are. *When Jeff reaches out to the bottom drawer and puts on a shirt, he believes the shirt is pink.*	yes

Appendix 3. Theory of Mind task vignettes.
